# Influence of droplet size on the antibacterial efficacy of citral and citronella oil nanoemulsions in polysaccharide coated fresh-cut apples

**DOI:** 10.1038/s41598-023-37528-9

**Published:** 2023-06-28

**Authors:** Mounir Touayar, Rania Zayani, Chokri Messaoud, Hesham Salman

**Affiliations:** 1 Bionanoplus, Polígono, C. E, N°2, 2°B, 31194 Orikain, Navarra Spain; 2 Research Unit of Nanobiotechnology and Valorisation of Phytoressources Medicinal Plants UR17ES22, National Institute of Applied Science and Technology, University of Carthage, Centre Urbain Nord, BP 676, 1080 Charguia Cedex, Tunisia

**Keywords:** Biotechnology, Microbiology

## Abstract

Fresh-cut fruits are highly perishable and susceptible to bacterial contamination. Polysaccharides edible coating loaded with essential oils nanoemulsions have the potential to extend shelf life and improve quality of fruits. The effectiveness of this approach is dependent on the properties of the nanoemulsions, such as droplet size (DS) and stability. This study aimed to optimize the production of citral (CT) and citronella oil (CTO) nanoemulsions (CT-CTO-NEs) incorporated in edible coating film to be used as natural antimicrobial agent in fresh-cut apples. After testing different combinations of surfactant (tween 80) and cosurfactant (propylene glycol) to obtain stable oil-in-water (o/w) nanoemulsions, the results demonstrated that optimized CT-CTO-NEs with DS less than 500 nm have been successfully achieved with high stability for 3 weeks at 4 °C. In addition, CT-CTO-NEs were obtained by In situ formation under magnetic stirring without applying complex high shear homogenization processes. Desired stability of CT-CTO-NEs also has been achieved within semi-solid matrix (sodium alginate cross-linked film). The relationship between DS and antibacterial activity was observed, with the smallest DS (< 100 nm) showing the highest antibacterial efficacy against *Listeria monocytogenes* and *Escherichia coli*. These results emphasize the importance of DS in the effectiveness of CT-CTO-NEs as an antibacterial coating for fresh-cut fruits.

## Introduction

Fresh-cut fruits have become popular among consumers due to their convenience and the increasing demand for minimally processed foods. Edible coating loaded with antimicrobial EOs is a promising strategy and an alternative to chemical preservatives to protect fresh-cut fruits from frequent contamination by pathogenic microorganisms^[1,2]^. The antimicrobial efficacy of some EOs such as CT, a major compound of lemongrass oil, and CTO have been reported against diverse bacteria such as *E. coli* and *L.* monocytogenes^[3–6]^. An effective tool for enhancing the antimicrobial efficacy of EOs is the use of oil in water (o/w) nanoemulsion technology, where EOs are optimally dispersed as small stabilized oil droplets in an aqueous phase using appropriate surfactants. Furthermore, compared to traditional emulsions, nanoemulsions increase the dispersibility of EOs within microbial surfaces, leading to a significant enhancement of their antimicrobial efficacy^[7–10]^. Additionally, it has been described that small droplets of EOs nanoemulsion interact with the phospholipid bilayer of microbial cell membranes, leading to an increase in the antibacterial efficacy^[11]^.

However, the choice of suitable combination between surfactants and/or cosurfactants used to obtain o/w nanoemulsions is a critical parameter that can influence the initial oil DS and stability during storage. Sedaghat Doost et al.^[11]^ highlights in his review the effect of different surfactants on the main physicochemical properties and the antimicrobial efficacy of EOs. The destabilization of this delivery system such as DS growth and phase separation may occur over time particularly if the components of the EOs have high water solubility and mobility due to Ostwald ripening^[12,13]^.

On the other hand, sodium alginate is a commonly used polysaccharide as a carrier (water phase in o/w nanoemulsion) of antimicrobial EOs to enhance the shelf-life of fruits such as apples. However, the use of alginate can lead to a change in the characteristics of the nanoemulsions due to micro fluidization process, which can negatively affect the nanoemulsions stability and its antimicrobial attributes^[14]^. Thus, the main challenge is to achieve stable o/w nanoemulsions with DS in the nano-range that remain stable over an extended period in edible coatings applied to fruits.

The DS of nanoemulsions is known to have an impact on its antimicrobial efficacy, but the relationship between DS and its functionality has not been deeply investigated. For this purpose, this work aims to optimize the ratio between edible surfactant and cosurfactant combinations to obtain highly stable CT and CTO nanoemulsions in sodium alginate film. Then, the influence of DS on the antibacterial efficacy of the EOs nanoemulsions will be assessed against *E. coli* and *L. monocytogenes* in coated fresh-cut apples.

## Materials and methods

### Materials

Sodium alginate was supplied from Ceamsa (Spain) (Food Grade:FG; medium viscosity 400 mPas) and it was the primary ingredient in all coating solutions. Calcium chloride, and Propylene glycol (USP Grade) were purchased from Guinama (Spain). Citral was provided by Sigma-Aldrich (Spain) (purity ≥ 96%, FG), citronella oil was purchased from Fagron Iberica (Spain) and it consisted mainly of geraniol (43.22%-45.02%), citronellal (11.65%-17.13%), citronellol (7.05%-8.14%), geranial (7.98%-12.63%), neral (6.01%-8.45%), geranyl acetate (4.22%-5.45%) and elemol (1.22%-5.98%). Tween 80 (USP) was purchased from Panreac (Spain). *Escherichia coli* (*E. coli,* 516 strain 11.1) provided by the University of Barcelona and *Listeria monocytogenes* (*L. monocytogenes*, 911 strain 21.1) from the Spanish Type Culture Collection (Spain), were used in this study. Finally, Growth media Tryptone Soy Agar (TSA) and Peptone were purchased from Panreac.

### Preparation of CT and CTO o/w nanoemulsions

CT and CTO have been selected in order to prepare EOs o/w nanoemulsions as antibacterial agents and their final concentrations have been chosen according to previous internal studies (minimum inhibitory concertation) for both selected pathogens in this study (data not shown).

In order to optimize CT and CTO nanoemulsions (CT-CTO-NEs), Surfactant tween 80 (TW 80) and cosurfactant propylene glycol (PG) were mixed with different weight ratios, named as surfactant-cosurfactant mixes (S-COS-Mix). Then, 5 g of CT-CTO (1:1 weight ratio) were incorporated into 95 g of different S-COS-Mix achieving final concertation of CT-CTO of 5% w/w. Finally, different S-COS-Mix with CT-CTO mixtures were added to sodium alginate solution at 1.5% w/v under magnetic stirring 300 rpm for 5 min to prepare CT-CTO-NEs, achieving final concentrations of CT-CTO of 0.05 and 0.1% w/w as illustrated in Table 1.

**Table 1:** Factors and modalities of factorial complete design.

Factor (K)	Modalities							
X1: % (S-COS-Mix)	0 (-1)	10 (0)	25 (1)	40 (2)	60 (3)	75 (4)	90 (5)	100 (6)
X2: % CT-CTO	0.05 (− 1)				0.1 (1)			

K = Retained factors (X1, X2), X1 = % S-COS-Mix: 8 modalities (%TW 80 and PG w/w), X2 = % CT-CTO: 2 modalities (% 0.05 and 0.1 w/w), N_Total_ = 2^1^ × 8^1^ = 16 combinations.

### Characterization and stability of CT-CTO-NEs incorporated in sodium alginate solution and cross-linked film

The freshly prepared CT-CTO-NEs were characterized by measuring oil DS and polydispersity index (PDI) using the Dynamic Light Scattering technique (DLS) and a particle size analyzer (90S, Brookhaven). The stability of the CT-CTO-NEs formulations was studied by monitoring changes in DS (nm) over 3 weeks at 4 °C in both sodium alginate solution and cross-linked film (Table 2). For this purpose, cross-linked sodium alginate films loaded with CT-CTO-NEs were prepared by adding 4 g of CT-CTO-NEs to cover a 10 cm^2^ area in a glass petri dish. Then, 0.3 mL of calcium chloride (2% w/v in distilled water) was sprayed onto the surface of the alginate layer. In order to ensure complete crosslinking, samples were left closed at room temperature for 5 h.

**Table 2:** Asymmetric experimental matrix of CT-CTO-NEs stability in sodium alginate solution and cross-linked film.

Experimental	Name	X1	X2	PDI	DS
1	CT-CTO-NE1	− 1	− 1	PDI 1	DS 1
2	CT-CTO-NE2	0	− 1	PDI 2	DS 2
3	CT-CTO-NE3	1	− 1	PDI 3	DS 3
4	CT-CTO-NE4	2	− 1	PDI 4	DS 4
5	CT-CTO-NE5	3	− 1	PDI 5	DS 5
6	CT-CTO-NE6	4	− 1	PDI 6	DS 6
7	CT-CTO-NE7	5	− 1	PDI 7	DS 7
8	CT-CTO-NE8	6	− 1	PDI 8	DS 8
9	CT-CTO-NE9	− 1	+ 1	PDI 9	DS 9
10	CT-CTO-NE10	0	+ 1	PDI 10	DS 10
11	CT-CTO-NE11	1	+ 1	PDI 11	DS 11
12	CT-CTO-NE12	2	+ 1	PDI 12	DS 12
13	CT-CTO-NE13	3	+ 1	PDI 13	DS 13
14	CT-CTO-NE14	4	+ 1	PDI 14	DS 14
15	CT-CTO-NE15	5	+ 1	PDI 15	DS 15
16	CT-CTO-NE16	6	+ 1	PDI 16	DS 16

Experimental: Different freshly prepared CT-CTO-NEs in sodium alginate solution and cross-linked film.

*PDI* Polydispersity index of freshly prepared CT-CTO-NEs in sodium alginate solution and cross-linked film, *DS* droplet size of freshly prepared CT-CTO-NEs in sodium alginate solution and cross-linked film. *CT-CTO-NE1-8* Final concentration of CT-CTO at 0.05% w/w, *CT-CTO-NE9-16* Final concentration of CT-CTO at 0.1% w/w.

The films were then washed with distilled water and stored in closed containers at 4 °C. The stability of CT-CTO-NEs in alginate solution was studied by weighing 4 g of each formulation in 20 mL crystal bottles and stored under the same conditions. Changes in DS were measured at 0, 1, 2, and 3 weeks. For this purpose, cross-linked films were dissolved in 4 mL sodium citrate solution (0.2% w/v) for 2 h, then the DS was determined as described above.

### Optimization of different CT-CTO-NEs by response surface methodology (RSM)

The optimization of different CT-CTO-NEs in both sodium alginate solution and cross-linked film was performed using response surface methodology (RSM) through statgraphics Centurion, version 18. Traditionally, experiments are carried out by randomly varying the factors one after the other and without conducting experiments with prior planning. The major drawback of this traditional methodology of a single study parameter variation is neglecting the interaction effect of the two factors at the same time. Thereby, restoring the use of a complete factorial experimental design with 16 combinations (8^1^ × 2^1^) was the solution. In fact, this method combines a thorough and complete way of all the possible combinations of the selected factor modalities.

The responses measured are DS and PDI of freshly prepared CT-CTO-NEs in sodium alginate solution and cross-linked film. The retained factors (K) represent the weight ratios of TW 80 and PG (S-COS-Mix in percentages; X1), as well as the final concentration of CT-CTO w/w (X2). The fixed factors modalities and experimental matrix are all illustrated in Tables 1 and 2.

### Fresh-cut apples coating process

For the film coating process, “Golden manzana” (Malus domestica) was purchased from a local market in the north of Spain and stored at 4 °C before processing. Sodium alginate (1.5% w/v) and calcium chloride (2% w/v) solutions were autoclaved at 121 °C for 20 min to ensure complete sterilization before being used in the elaboration of nanoemulsions and coating process.

The apples were sanitized in a 200 ppm sodium hypochlorite solution prior to cutting. The coating process was performed by dipping apple pieces during 2 min into alginate coating solutions containing CT-CTO-NEs formulations as described below. Then, samples were left 2 min to drip off the coating solution excess before dipping samples in calcium chloride at 2% w/v for 2 min.

### Antibacterial efficacy of different CT-CTO-NEs incorporated into sodium alginate cross-linked film in fresh-cut apples

#### Inoculum preparation and fresh-cut apples inoculation

For the preparation of the bacterial inoculum, TSA was used as growth medium for both of *E. coli* and *L. monocytogenes* and they were grown during 24 h at 37 °C. Then, Microorganisms’ concentration was adjusted to $10^{6}$ CFU/mL using saline peptone water at 0.1% w/v^[15]^.

In order to check the DS influence on the antibacterial efficacy of the nanoemulsions in fresh-cut apples, all CT-CTO-NEs with DS below 500 nm during 3 weeks at 4 °C, have been considered stable and were incorporated into sodium alginate cross-linked film. For this purpose, apple samples were cut into equal slices of 1 cm^2^ × 1 cm in length under aseptic conditions and coated as previously described. Then, coated samples were inoculated with 10 µL of diluted inoculum already prepared at 10^6^ CFU/mL. Finally, all samples were packaged into a sterile bag (Poly propylene bags), dried under aseptic conditions at room temperature, and stored at 4 °C.

#### Antibacterial efficacy assay

The bacterial count of cut apples inoculated with both selected microorganisms was evaluated at days 0, 3, 7, 14, and 21 in triplicate. From each sample, 0.1 g of apple was diluted in 0.9 mL of sterile Phosphate buffered saline (PBS, 10 mM) smashed with a sterile spatula in Eppendorf tubes and homogenized for 1 min with vortex. The microbial count was performed by plating serial dilutions on TSA plates for 24 h at 37 °C. Finally, visible colonies were counted and expressed as CFU/g of cut apple as described by Song et al.^[16]^.

### Statistical analysis

Finally, all tests were applied in triplicates and results were expressed as mean ± standard deviation (SD). Statistical differences among experimental data were determined by analysis of variance (ANOVA) using SPSS software (SPSS 15.0). The evaluation of significant differences among values was applied by means of Duncan’s multiple tests at a level of *p* < 0.05.

### Compliance with requested guidelines

All plant studies involving “Golden manzana” (Malus domestica) were carried out in accordance with relevant institutional, national, and international guidelines and legislation.

## Results and discussion

### Characterization of different CT-CTO-NEs incorporated into sodium alginate solution and cross-linked film

#### Droplet size (DS) and polydispersity index (PDI) analysis of different CT-CTO-NEs freshly prepared in sodium alginate solution and cross-linked film

The DS and PDI of freshly prepared CT-CTO-NEs in sodium alginate solutions and cross-linked films were characterized through the Dynamic Light Scattering technique (DLS) and the particle size analyzer (90S, Brookhaven). Results in Table 3 demonstrate that when the final concentration of CT-CTO was 0.05% w/w (in CT-CTO-NE1,2,3,4,5,6,7 and 8), smaller DS were obtained at all weight ratios of S-COS-Mix (Table 2) compared to CT-CTO-NE9,10,11,12,13,14,15 and 16 (CT-CTO final concertation at 0.1% w/w).

**Table 3:** DS and PDI of freshly prepared CT-CTO-NEs in sodium alginate solution and cross-linked film.

Formulations	CT-CTO-NEs DS (nm) in sodium alginate solution Mean ± SD	PDI Mean ± SD	CT-CTO-NEs DS (nm) in sodium alginate cross-linked film Mean ± SD	PDI Mean ± SD
CT-CTO-NEs 1	931 ± 145^i^	0.38 ± 0.10^def^	1136 ± 103^j^	0.32 ± 0.13^efgh^
CT-CTO-NEs 2	833 ± 36^g^	0.32 ± 0.03^de^	978 ± 35^i^	0.42 ± 0.15^gh^
CT-CTO-NEs 3	254 ± 23^c^	0.08 ± 0.09^ab^	262 ± 18^cd^	0.13 ± 0.02^abcd^
CT-CTO-NEs 4	60 ± 13^a^	0.06 ± 0.02^a^	58 ± 10^a^	0.08 ± 0.02^a^
CT-CTO-NEs 5	180 ± 24^b^	0.10 ± 0.03^ab^	193 ± 13^b^	0.12 ± 0.03^abc^
CT-CTO-NEs 6	440 ± 19^d^	0.07 ± 0.05^ab^	456 ± 21^e^	0.07 ± 0.01^a^
CT-CTO-NEs 7	735 ± 18^f^	0.26 ± 0.04^ cd^	752 ± 22^g^	0.23 ± 0.03^bcdef^
CT-CTO-NEs 8	866 ± 10^gh^	0.41 ± 0.12^ef^	998 ± 18^i^	0.32 ± 0.15^fgh^
CT-CTO-NEs 9	1108 ± 106^j^	0.46 ± 0.12^ef^	1213 ± 94^k^	0.29 ± 0.1^defg^
CT-CTO-NEs 10	954 ± 84^i^	0.42 ± 0.1^ef^	989 ± 29^i^	0.32 ± 0.16^fgh^
CT-CTO-NEs 11	388 ± 38^d^	0.08 ± 0.05^ab^	302 ± 22^d^	0.15 ± 0.06^abcd^
CT-CTO-NEs 12	98 ± 10^a^	0.04 ± 0.02^a^	112 ± 9^a^	0.08 ± 0.03^a^
CT-CTO-NEs 13	189 ± 28^bc^	0.06 ± 0.04^ab^	225 ± 16^bc^	0.18 ± 0.05^abcde^
CT-CTO-NEs 14	630 ± 24^e^	0.18 ± 0.08^bc^	595 ± 32^f^	0.1 ± 0.03^ab^
CT-CTO-NEs 15	910 ± 16^hi^	0.36 ± 0.04^def^	868 ± 36^h^	0.26 ± 0.02^cdef^
CT-CTO-NEs 16	1224 ± 18^k^	0.48 ± 0.1^f^	1112 ± 21^j^	0.45 ± 0.1^h^

Values are means ± SD; the values followed by the same lower-case letter, in the same column and parameter, and by the same upper-case letter in the same row are not significantly different (Duncan’s new multiple range test at *p* < 0.05).

Furthermore, CT-CTO-NE3,4,5 and 6 showed a homogenous DS below 500 nm and PDI lower than 0.2. However, CT-CTO-NE1,2,7 and 8 showed bigger and more heterogenous DS and PDI values higher than 0.2.

On the other hand, the smallest DS (98–388 nm) and the lowest PDI values (ranging from 0.04–0.08), while incorporating CT-CTO at 0.1% w/w, were observed in CT-CTO-NE11,12 and 13, indicating a homogenous size distribution compared to CT-CTO-NE9,10,14 and 15 that showed a higher DS and PDI ranging from 0.18 to 0.48.

**Figure 1:**
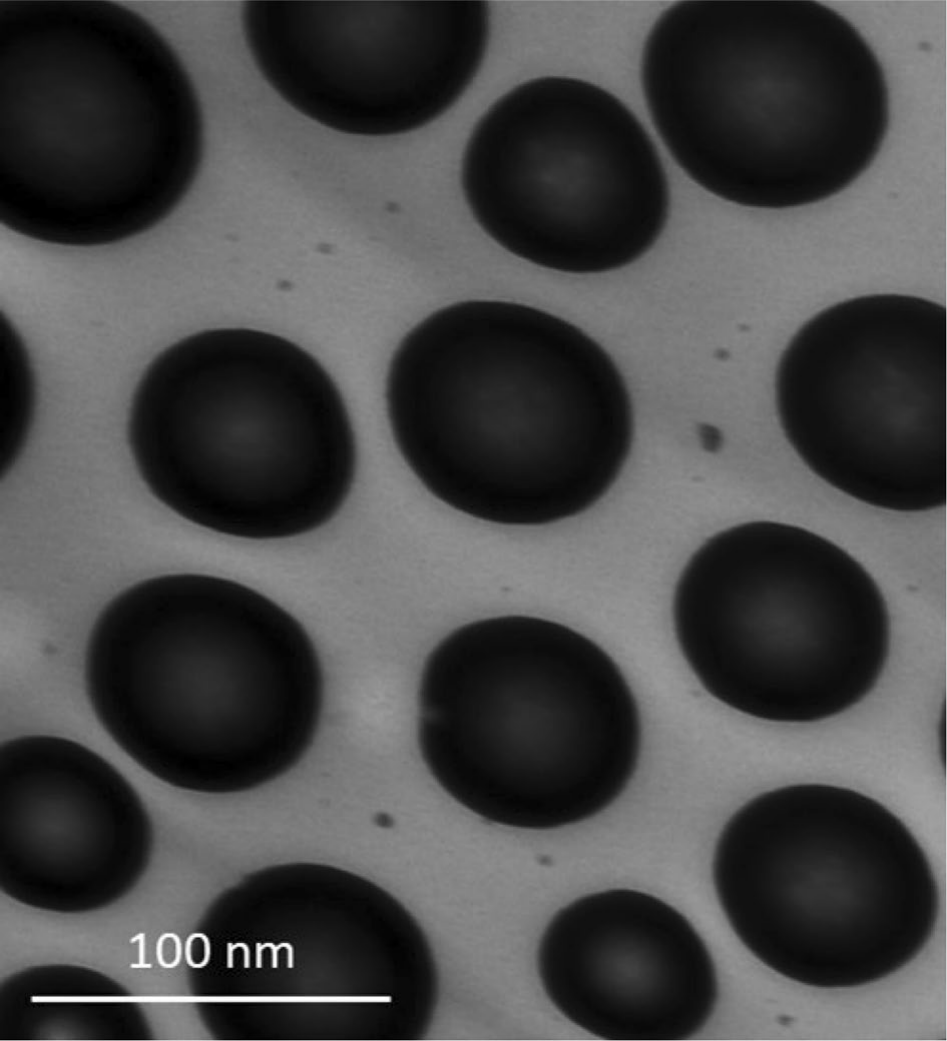
TEM: morphological characterization of CT-CTO-NE4. Image of scale bar 100 nm at 64,000 magnifications.

In Fig. 1, the use of transmission electron microscope (TEM) Zeiss Libra® 120 (Oberkochen, Germany) at 640,000 magnifications shows perfectly the homogeneous dispersion of CT-CTO-NE4 droplets with a DS below 100 nm.

The differences observed in DS and PDI of different CT-CTO-NEs prepared in this work indicated the importance of using adequate combinations between surfactant (TW 80) and cosurfactant (PG) to obtain small DS. Generally, mixing TW 80 and PG allowed to achieve smaller DS and homogenous nanoemulsions. However, the use of PG or TW 80 separately showed heterogenous and higher DS nanoemulsions (PDI values in Table 3).

Previous studies have reported that the absence of cosurfactants in the production of EOs nanoemulsions can result in obtaining small DS but with heterogeneous distributions (PDI > 0.2) when using high shear processing methods such as Ultra Turrax or microfluidization^[17]^. In fact, Gago et al.^[17]^ reported the manufacture of EOs nanoemulsions using a sodium alginate solution at 2% and a single surfactant (TW 80) via microfluidization. Despite obtaining small DS in their nanoemulsions, their PDI values were significantly higher than those obtained in our study. Additionally, high pressure microfluidization was needed to achieve small DS in their system, while our results showed similar DS without using this technique. Similarly, Pitrozzi et al.^[18]^ described a strategy to improve the shelf-life of tomato by using edible coatings with sodium alginate cross-linked film using oregano essential oil nanoemulsion. In this work, high shear processing (Ultra Turrax) was applied to obtain DS around 176.8 nm and a PDI of 0.26, when only lecithin was used as a surfactant.

The combination of surfactant and cosurfactant has been previously described to obtain highly stable pine oil-loaded nanoemulsions using non-ionic surfactant (Tw 80) and cosurfactant (Volatile organic solvent: Ethanol) for the oral delivery of nutraceutical^[19]^. This study demonstrates the ability to achieve small DS without requiring the implementation of complex high shear homogenization techniques.

#### Optimization of different CT-CTO-NEs by response surface methodology (RSM)

The optimization of different CT-CTO-NEs formulations in sodium alginate solution and cross-linked film was performed using response surface methodology (RSM) through statgraphics by following complete factorial experimental design with 16 combinations (8^1^ × 2^1^), which combine all possible combinations of the selected factors and modalities exhaustively and comprehensively.

The obtention of minimal DS of different CT-CTO-NEs in sodium alginate solution and cross-linked film can be achieved by determining the appropriate weight ratio of TW 80 and PG (S-COS-Mix) and CT-CTO final concentration in both coating solution and cross-linked film.

Tables 4 and 5 illustrate the combinations of factor levels used to minimize the DS and PDI responses in sodium alginate solution and cross-linked film. The R^2^ values indicate that the obtained models explain 77.2263–93.3799% of the variability of the responses. This analysis showed that all responses present an R^2^ value higher than 90%, except for the CT-CTO-NEs PDI in sodium alginate cross-linked film (77.2263%).

**Table 4:** DS and PDI of freshly prepared CT-CTO-NEs in sodium alginate solution.

Response	Model suitability (R^2^) (%)	Model equation	Optimal (w/w)	Optimal value
PDI	92.0578	$$\begin{aligned} {\text{PDI}} & = 0.388934 - 0.0168642 \times {\text{X}}1 \times 0.0787565 \times {\text{X}}2 \\ & \quad + \;0.00016574 \times {\text{X}}1^{2} + 0.00042487 \times {\text{X}}1{\text{X}}2 \\ \end{aligned}$$	X1 = 50.87% X2 = 0.03%	0.04
DS	93.3799	$$\begin{aligned} {\text{DS}} & = 957.682 + 1795.82 \times {\text{X}}2 - 39.5736 \times {\text{X}}1 \\ & \quad + \;15.5337 \times {\text{X}}1{\text{X}}2 + 0{ }.391601 \times {\text{X}}2^{2} \\ \end{aligned}$$	X1 = 49.53% X2 = 0.05%	86.54 nm

**Table 5:** DS and PDI of freshly prepared CT-CTO-NEs in sodium alginate cross-linked film.

Response	Model suitability (R^2^)	Model equation	Optimal (w/w)	Optimal value
PDI	77.2263%	–	–	–
DS	93.1691%	$$\begin{aligned} {\text{DS}} & = 1158.2 - 43.1033 \times {\text{X}}1 + 59.6878 \times {\text{X}}2 \\ & \quad + \;0.412334 \times {\text{X}}1^{2} + 1.72124 \times {\text{X}}1{\text{X}}2 \\ \end{aligned}$$	X1 = 52.26% X2 = 0.05%	31.75 nm

**Figure 2:**
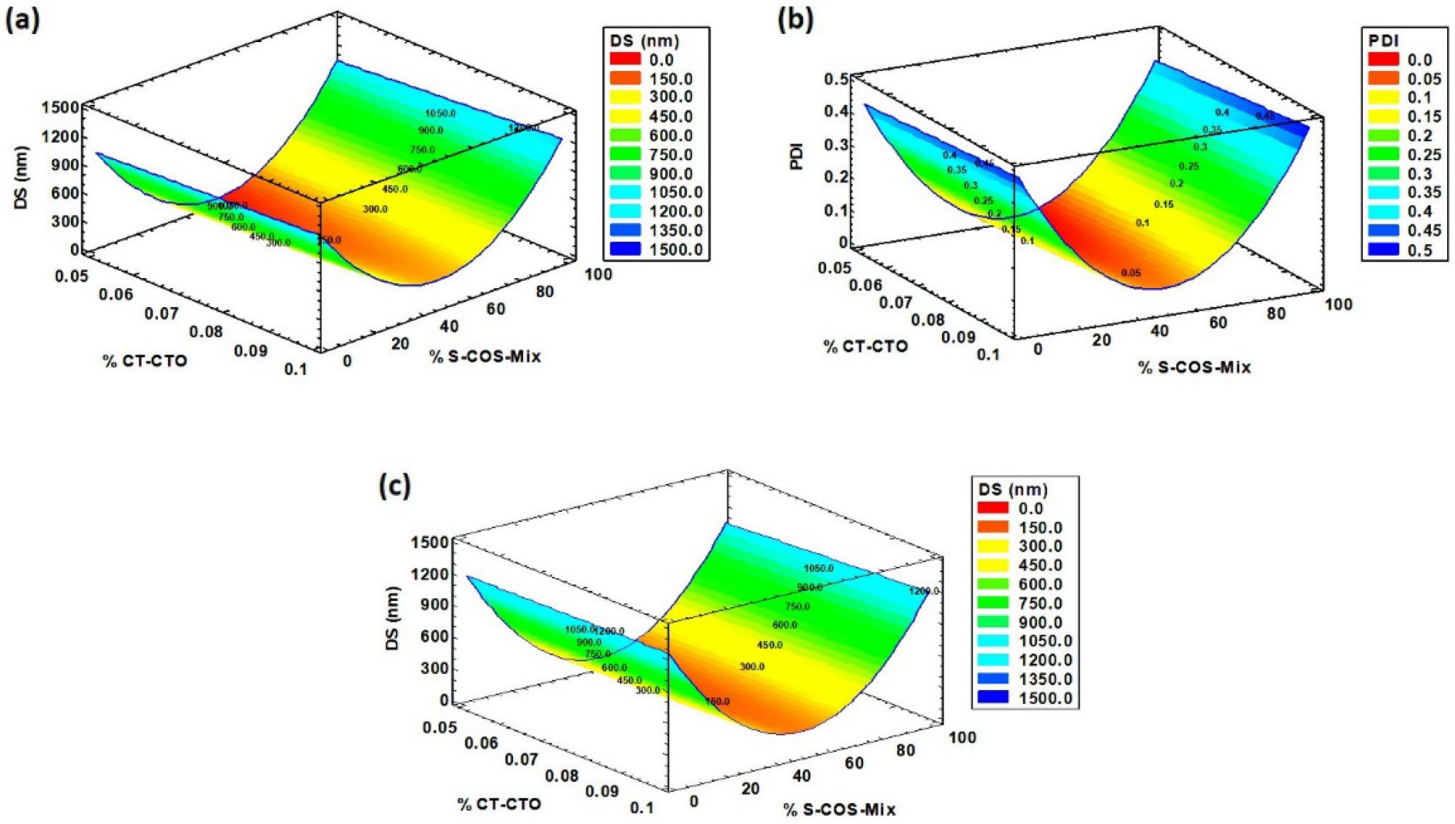
Response surfaces of DS and PDI of freshly prepared CT-CTO-NEs in sodium alginate solution and cross-linked film. (**a**, **b**) in sodium alginate solution, (**c**) in sodium alginate cross-linked film.

The establishment of equations for each response allowed the determination of the optimal experimental conditions for minimizing the DS and PDI of freshly prepared CT-CTO-NEs in a sodium alginate solution and cross-linked film (Tables 4, 5). The analysis of the different response surfaces (Fig. 2) showed that the selected factors had a significant effect on both the DS and PDI.

The optimal DS in sodium alginate solution (86.54 nm) was achieved with TW 80 at around 49.53% and a CT-CTO final concentration of 0.05% (Table 4) and optimal PDI was achieved at 50.87% of TW 80 and a CT-CTO final concentration of 0.03% (Table 4). Additionally, the optimal DS in sodium alginate cross-linked film (31.75 nm) was achieved using TW 80 at around 52.26% and a CT-CTO final concentration of 0.05% w/w (Table 5).

The combination between TW 80 and PG in ratios ranging from 25:75 to 75:25 w/w seems to be the most adequate ratio to obtain particle sizes below 500 nm in both sodium alginate solution and cross-linked film when CT-CTO was incorporated at 0.05% w/w. This confirms the results estimated by the optimization model used through RSM (Fig. 2). Furthermore, the smallest DS was observed when TW 80 was used at a ratio of approximately 40 to 60 w/w with PG when CT-CTO was incorporated at 0.05% w/w. The results in the present work did not show a significant difference from the predicted optimal values. The smallest DS achieved for CT-CTO-NEs in sodium alginate solution was only 60 nm compared to 69.16 nm (predicted optimal value). Similarly, in the sodium alginate cross-linked film, the obtained DS was 58 nm compared to 86.54 nm (predicted optimal value).

#### DS change of different CT-CTO-NEs in sodium alginate solution and cross-linked film

**Figure 3:**
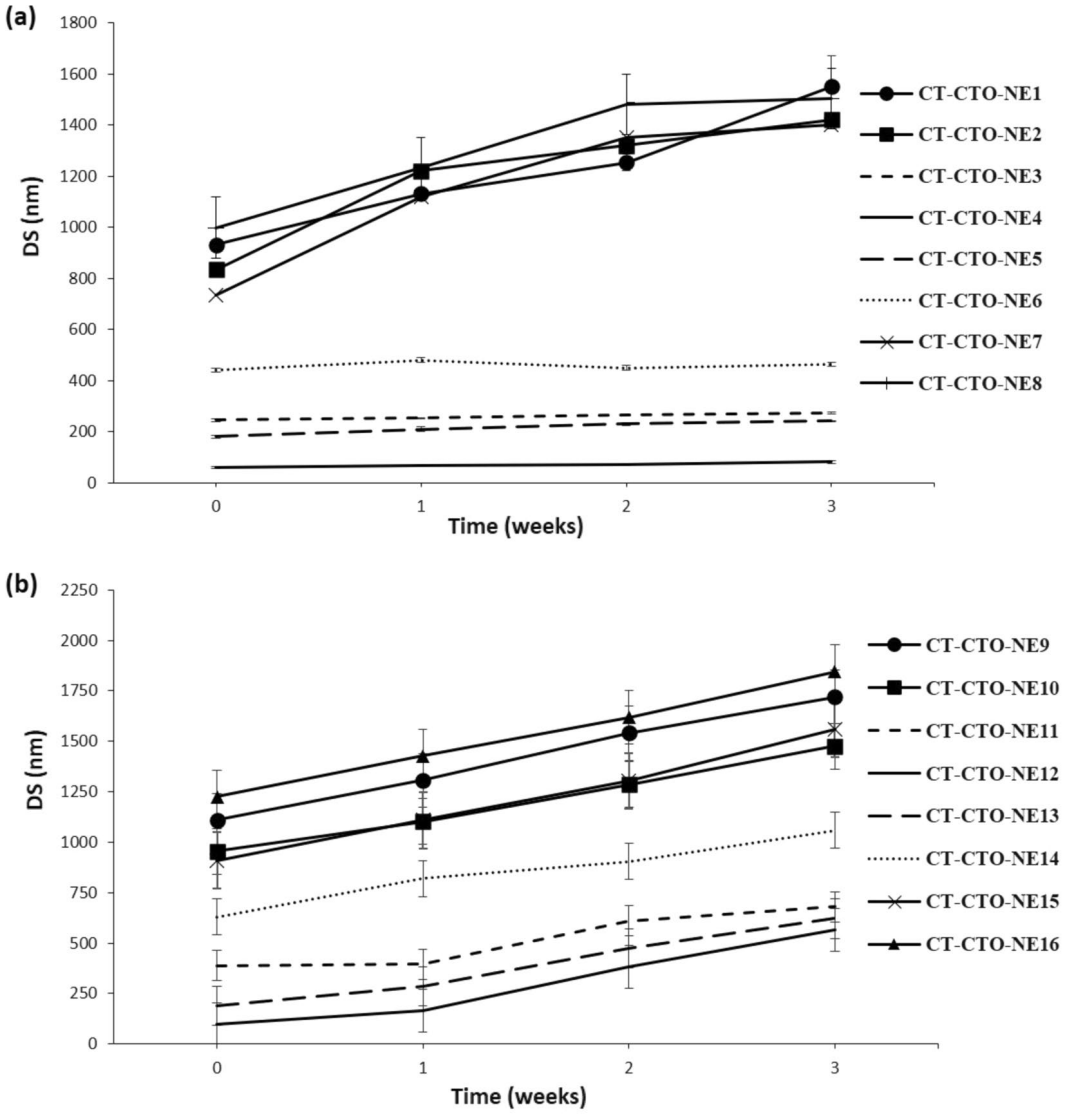
DS change of different CT-CTO-NEs in sodium alginate solution during 3 weeks at 4 °C; (**a**) final concentration of CT-CTO 0.05% w/w, (**b**) final concentration of CT-CTO 0.1% w/w.

**Figure 4:**
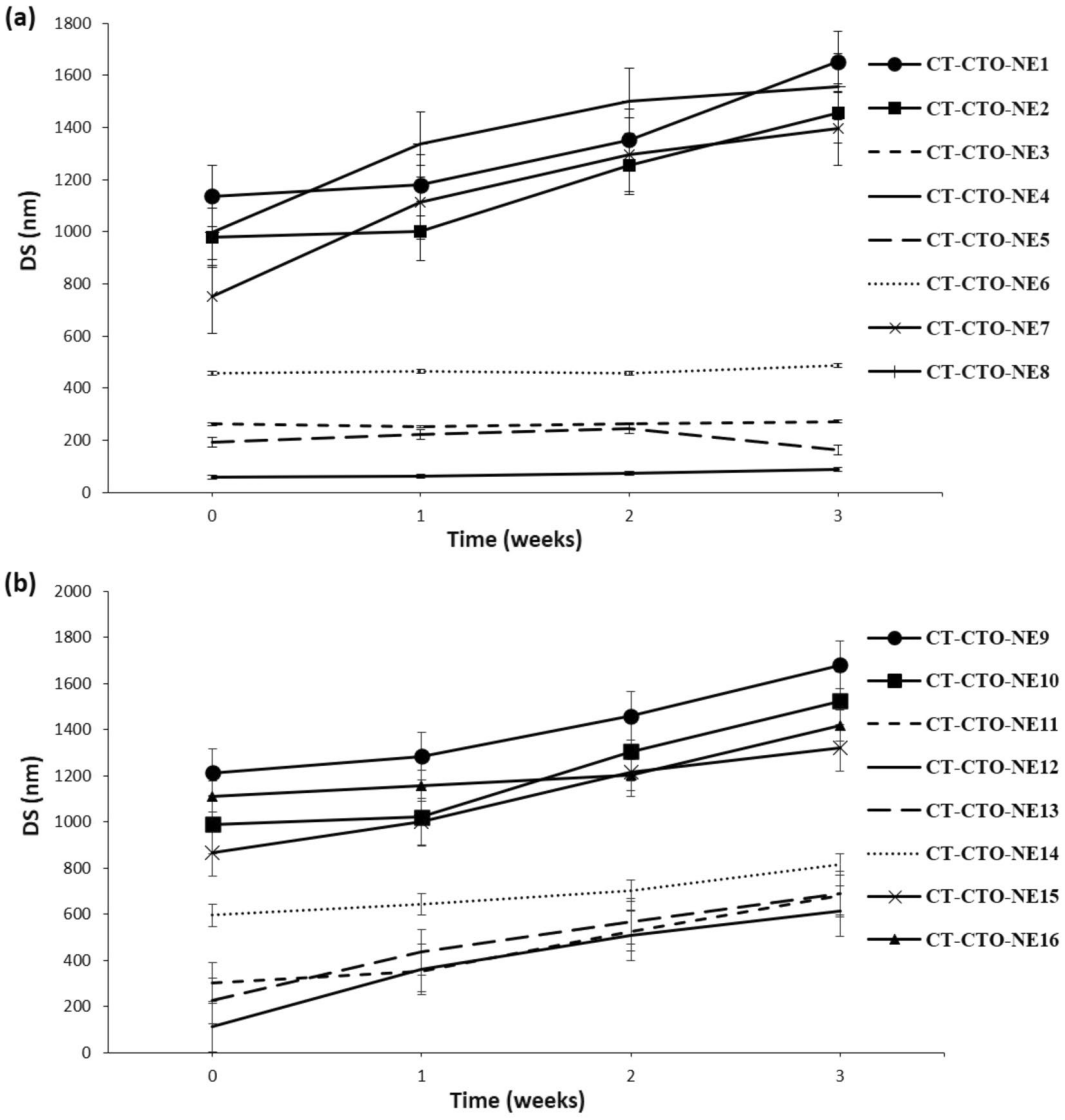
DS change of different CT-CTO-NEs in sodium alginate cross-linked film during 3 weeks at 4 °C; (**a**) final concentration of CT-CTO 0.05% w/w, (**b**) final concentration of CT-CTO 0.1% w/w.

The change in DS during 3 weeks at 4 °C of different CT-CTO-NEs was assessed in both sodium alginate solution and cross-linked film. Results in Figs. 3a and 4a illustrate the stability of different CT-CTO-NEs when CT-CTO was incorporated at a final concentration of 0.05% w/w in sodium alginate solution and cross-linked film. It can be clearly observed that CT-CTO-NE1,2,7 and 8 were unstable due to a significant increase in DS along the experiment.

On the other hand, CT-CTO-NE3,4,5 and 6 did not show significant changes in DS during 3 weeks in both sodium alginate solution and cross-linked film. Interestingly, CT-CTO-NE4 was the most stable formulation, with a very low DS change (between 4–14 nm) during the storage period.

Results shown in Figs. 3b and 4b demonstrate that, when CT-CTO was incorporated at a final concentration of 0.1% w/w using the same S-COS-Mix combinations, higher changes in DS were observed. Thus, at the end of the storage period, all samples with significant change in DS (*p* < 0.05) were considered unstable.

The results found in this study demonstrate that in order to obtain a stable EOs nanoemulsion in sodium alginate solution and cross-linked film using simple magnetic stirring, it is necessary to choose the optimal ratio between surfactant and cosurfactants (TW 80 and PG). However, increasing the final concentration of CT-CTO from 0.05% to 0.1% w/w in CT-CTO-NEs led to unstable formulations. This may be related to the use of mild magnetic stirring instead of applying high shear homogenization such as Ultra Turrax which is commonly used for the manufacturing of EOs nanoemulsions. Nevertheless, Pitrozzi et al.^[18]^ could not obtain DS less than 500 nm when only lecithin was used as a surfactant with high shear processing for oregano oil nanoemulsion elaboration at 0.5%. In addition, small DS and highly stable Citral, clove and lemongrass EOs nanoemulsions were obtained using only surfactant (TW 80), but microfluidization pressure (At 150 MPa for 5 cycles) was applied^[17]^. One of the challenges is related to the stability of DS within semi-solid matrix, herein cross-linked alginate film.

In fact, choosing the appropriate surfactant or surfactant-cosurfactant mixture ratios depends on many factors, such as the concentration of EOs volatile compounds, molecular structure, surfactant affinity, viscosity, and interfacial tension^[20]^. In addition, the surfactant-oil ratio plays an important role in the formation of nanoemulsions with different DS^[21]^.

### Antibacterial efficacy of sodium alginate cross-linked film loaded with selected CT-CTO-NEs in fresh-cut apples

According to the previous results in this research, CT-CTO-NEs 3,4,5 and 6 in sodium alginate cross-linked film with DS below 500 nm during storage period were selected for the antibacterial test.

**Figure 5:**
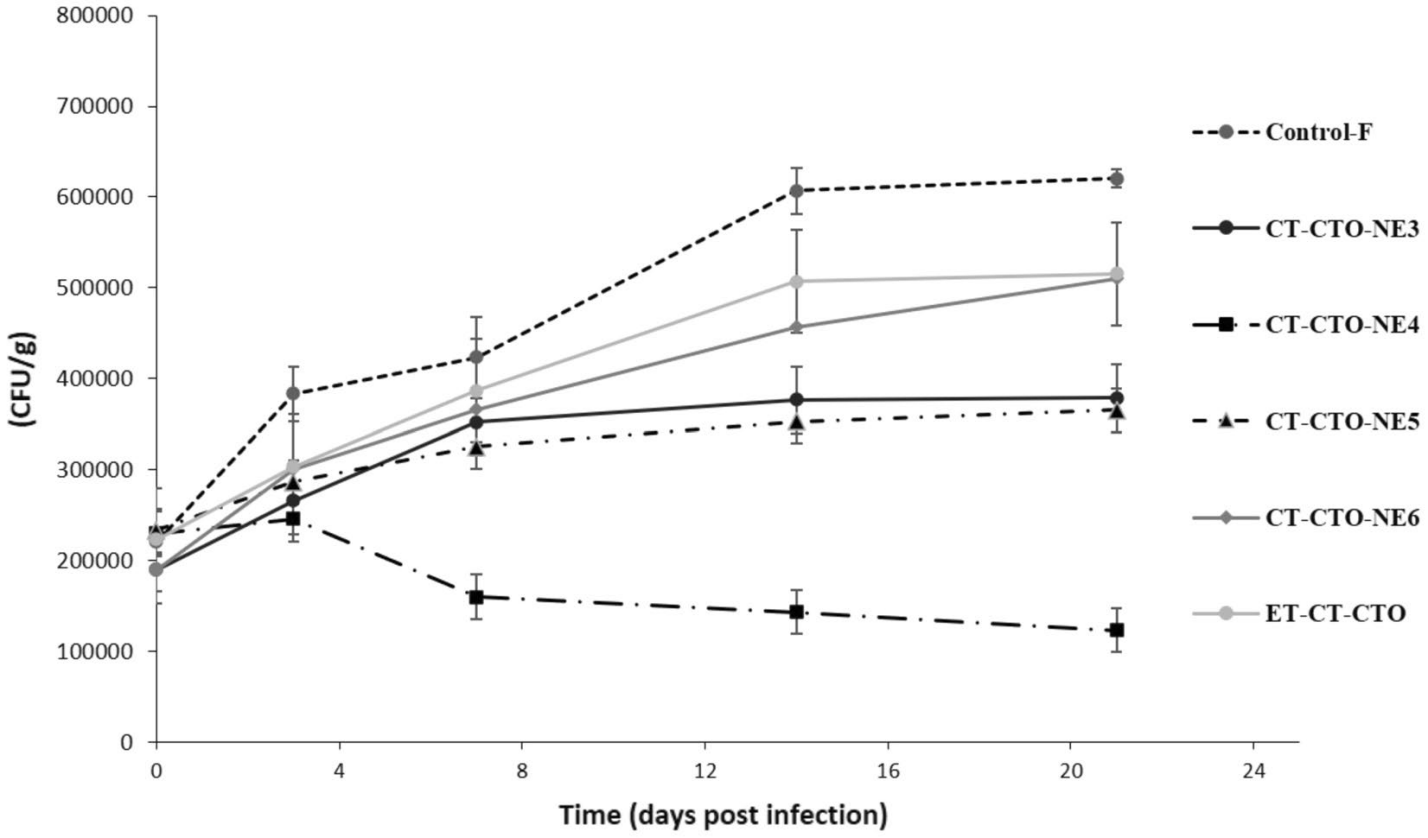
Antibacterial efficacy of selected formulations incorporated in sodium alginate cross-linked film against *L. monocytogenes*.

**Figure 6:**
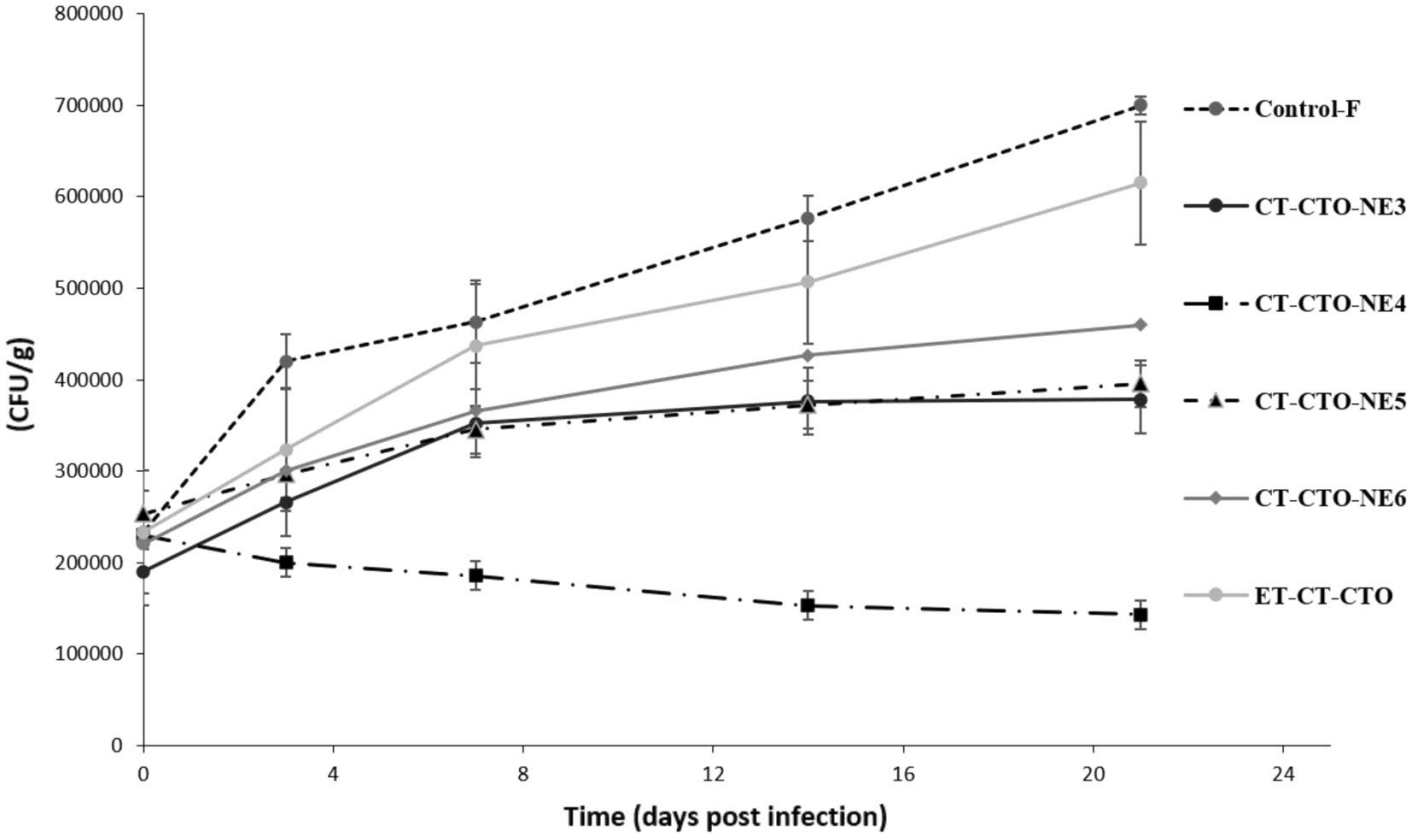
Antibacterial efficacy of selected formulations incorporated in sodium alginate cross-linked film against *E. coli*.

The antibacterial efficacy of CT-CTO-NE3,4,5 and 6 against *L*. *monocytogenes* and *E. coli* is presented in Figs. 5 and 6 as CFU/g. Fresh-cut apples coated only with sodium alginate cross-linked film without CT-CTO (negative control) showed a significant growth of approximately two to three times more than the initial bacterial count during 21 days. In case of ethanol-based formulations (ET-CT-CTO) no significant difference was observed compared to the negative control (*p* ˃ 0.05). These results indicate that the evaporation of ethanol may lead to phase separation and EOs high instability.

Regarding CT-CTO-NE6 (DS around 420 nm), a slightly better performance against *E. coli* than *L. monocytogens* was observed during 21 days compared to the negative control and ET-CT-CTO. In fact, the bacterial count registered for *E. coli* increased by two times while in case of *L. monocytogenes* it increased by almost three times compared to the initial count.

On the other hand, the efficacy of CT-CTO-NE 3 and 5 (DS around 200-220 nm) was similar, and both showed a minor enhancement in the inhibition of both microorganisms when compared with both controls (ET-CT-CTO and negative control). In this case, the decrease in DS from 420 (CT-CTO-NE6) to 200-220 nm (CT-CTO-NE 3 and 5) did not significantly enhance the antibacterial efficacy.

Interestingly, CT-CTO-NE4 (DS around 60 nm) showed the highest efficacy against both selected microorganisms. Thus, CT-CTO-NE4 presented a significant difference in antibacterial activity (*p* < 0.05) compared to both controls and the rest of formulations. This variation in antibacterial efficacy among different CT-CTO-NEs incorporated into sodium alginate cross-linked film may be attributed to the difference in DS. In fact, CT-CTO-NE4 represented the smallest DS and remained stable during 3 weeks of storage. Thereby, clearly explaining why the highest antibacterial efficacy was observed in CT-CTO-NE4 among all the tested formulations.

Incorporation of essential oils (EOs) into nanoemulsions and edible coatings has been found to effectively enhance the antibacterial attributes of these oils. Bhargava et al.^[22]^ demonstrated that the use of oregano oil in nanoemulsion at 0.05% applied on lettuce had a significant effect on *E. coli* and *L. monocytogenes* colonies reduction when compared to free oil. In a different study, the use of lemongrass oil nanoemulsion in sodium alginate edible coating film used to enhance the shelf-life of fresh-cut apples showed a higher antimicrobial efficacy against *E. coli* compared to free lemongrass oil^[23]^.

Although there is limited published work describing the use of CT and particularly CTO nanoemulsions as antibacterial agents, the variations in the compositions of natural EOs or possible synergy effect between CT and CTO make it difficult to compare with other published studies. Prakash et al.^[24]^ reported that the incorporation of CT nanoemulsion into alginate coating at concentrations of 0.1%, 0.5%, and 1% with DS ranging from 66.67 to 131.08 nm effectively reduced microbial growth in pineapple fruits. Additionally, their study suggested that increasing the concentration of CT nanoemulsion in alginate film displayed stronger antibacterial activity against *L. monocytogenes*.

However, Gago et al.^[17]^ demonstrated that the use of CT nanoemulsion at a concentration of 0.4%, with a DS of around 40 nm, which is eight times higher than the concentration of the CT and CTO mix used in our study, did not show any antibacterial effect against *E. coli*. Thus, the mixture of CT and CTO in a 1:1 ratio resulted in an increase in the antibacterial efficacy of CT at a lower concentration, indicating a synergistic effect.

The present study's findings are noteworthy due to their alignment with several prior investigations, while simultaneously contradictory with others, concerning the impact of DS on antibacterial activity. Prior research has shown that the smaller the DS is, the better the antibacterial activity may occur^[7,24,25]^. However, Terjung et al.^[26]^ found that larger droplets (3000 nm) exhibited higher antimicrobial efficacy than smaller droplets (200 nm) of carvacrol and eugenol against *E. coli* and *L. innocua*. Meanwhile, Buranasuksombat et al.^[27]^ claimed that the antimicrobial properties of nanoemulsions are primarily due to the active compounds in the emulsion, rather than the cell wall diffusion activity and high surface tension of droplets at the nano-scale.

## Conclusion

The present work has demonstrated that the use of edible coatings loaded with EOs-based nanoemulsions ensures an effective antibacterial activity on minimally processed apples.

The use of both TW 80 as a surfactant and PG as a cosurfactant in different combinations and ratios led to the obtention of different DS of CT-CTO-NEs in sodium alginate solution and cross-linked film when CT-CTO was incorporated at 0.05% and 0.1% w/w. In addition, high shear homogenization processes were not needed in order to obtain small DS below 500 nm with PDI values ranging between 0.04 and 0.08. Appropriate stabilities were achieved when CT-CTO was incorporated at 0.05% w/w in food coating matrix. Notably, CT-CTO-NE4 had the smallest DS during 3 weeks (≤ 89 nm) among all other formulations and exhibited the most significant growth inhibition of *E. coli* and *L. monocytogenes* on fresh-cut apple surfaces during all the experiment.

This study highlighted the correlation between the DS of CT-CTO-NEs and their antibacterial activity against *E. coli* and *L. monocytogenes* on fresh-cut apples. In fact, results demonstrated that nanoemulsions with smaller DS exhibited stronger antibacterial effects. The findings of this study provide valuable understandings into the optimization of edible coatings for preserving minimally processed fruits. These insights can have significant implications for developing more sustainable and efficient methods for ensuring the safety and quality of these products in the food industry.

## Data Availability

The data presented in this work are available on request from the corresponding author. The data are not publicly available due to being used in other future works.

## Abbreviations

CT: Citral
CTO: Citronella oil
CT-CTO-NEs: Citral-citronella oil nanoemulsion
DS: Droplet size
TW 80: Tween 80
PG: Propylene glycol
EOs: Essential oils
PDI: Polydispersity index
